# Inhibition of cAMP-Dependent PKA Activates β_2_-Adrenergic Receptor Stimulation of Cytosolic Phospholipase A_2_ via Raf-1/MEK/ERK and IP_3_-Dependent Ca^2+^ Signaling in Atrial Myocytes

**DOI:** 10.1371/journal.pone.0168505

**Published:** 2016-12-15

**Authors:** M. R. Pabbidi, X. Ji, J. T. Maxwell, G. A. Mignery, A. M. Samarel, S. L. Lipsius

**Affiliations:** 1 Department of Pharmacology, University of Mississippi Medical Center, Jackson, MS, United States of America; 2 Department of Physiology, Loyola University Medical Center, Maywood, IL, United States of America; 3 Department of Medicine, Loyola University Medical Center, Maywood, IL, United States of America; German Cancer Research Center (DKFZ), GERMANY

## Abstract

We previously reported in atrial myocytes that inhibition of cAMP-dependent protein kinase (PKA) by laminin (LMN)-integrin signaling activates β_2_-adrenergic receptor (β_2_-AR) stimulation of cytosolic phospholipase A_2_ (cPLA_2_). The present study sought to determine the signaling mechanisms by which inhibition of PKA activates β_2_-AR stimulation of cPLA_2_. We therefore determined the effects of zinterol (0.1 μM; zint-β_2_-AR) to stimulate I_Ca,L_ in atrial myocytes in the absence (+PKA) and presence (-PKA) of the PKA inhibitor (1 μM) KT5720 and compared these results with atrial myocytes attached to laminin (+LMN). Inhibition of Raf-1 (10 μM GW5074), phospholipase C (PLC; 0.5 μM edelfosine), PKC (4 μM chelerythrine) or IP_3_ receptor (IP_3_R) signaling (2 μM 2-APB) significantly inhibited zint-β_2_-AR stimulation of I_Ca,L_ in–PKA but not +PKA myocytes. Western blots showed that zint-β_2_-AR stimulation increased ERK1/2 phosphorylation in–PKA compared to +PKA myocytes. Adenoviral (Adv) expression of dominant negative (dn) -PKCα, dn-Raf-1 or an IP_3_ affinity trap, each inhibited zint-β_2_-AR stimulation of I_Ca,L_ in + LMN myocytes compared to control +LMN myocytes infected with Adv-βgal. In +LMN myocytes, zint-β_2_-AR stimulation of I_Ca,L_ was enhanced by adenoviral overexpression of wild-type cPLA_2_ and inhibited by double dn-cPLA_2_^S505A/S515A^ mutant compared to control +LMN myocytes infected with Adv-βgal. In–PKA myocytes depletion of intracellular Ca^2+^ stores by 5 μM thapsigargin failed to inhibit zint-β_2_-AR stimulation of I_Ca,L_ via cPLA_2_. However, disruption of caveolae formation by 10 mM methyl-β-cyclodextrin inhibited zint-β_2_-AR stimulation of I_Ca,L_ in–PKA myocytes significantly more than in +PKA myocytes. We conclude that inhibition of PKA removes inhibition of Raf-1 and thereby allows β_2_-AR stimulation to act via PKCα/Raf-1/MEK/ERK1/2 and IP_3_-mediated Ca^2+^ signaling to stimulate cPLA_2_ signaling within caveolae. These findings may be relevant to the remodeling of β-AR signaling in failing and/or aging heart, both of which exhibit decreases in adenylate cyclase activity.

## Introduction

We previously reported that attachment of atrial myocytes to the extracellular matrix protein laminin (LMN) acts via β_1_ integrin receptors to decrease β_1_-adrenergic receptor (AR) and increase β_2_-AR stimulation of L-type Ca^2+^ current (I_Ca,L_) [**1**]. Cell attachment to LMN decreases β_1_-AR signaling by inhibiting adenylate cyclase activity and diminishing cAMP levels via integrin-dependent activation of focal adhesion kinase (FAK)/phosphatidyinositol-3’ kinase (PI-3K)/protein kinase B (Akt) signaling [2]. We also reported that atrial cell attachment to LMN enhances β_2_-AR signaling by activating Gi/ERK/cytosolic phospholipase A_2_ (cPLA_2_)/arachidonic acid (AA) stimulation of I_Ca,L_ [**3**]. β_2_-AR activation of cPLA_2_ signaling is dependent on concomitant LMN-mediated inhibition of adenylate cyclase/cAMP-dependent kinase (PKA) [**3**]. In other words, cell attachment to LMN acts via inhibition of adenylate cyclase/PKA to both inhibit β_1_-AR signaling and enhance β_2_-AR signaling through activation of cPLA_2_. In embryonic chick ventricular myocytes [**4**] and rat ventricular myocytes [**5**] β_2_-AR stimulation also activates cPLA_2_/AA signaling. Moreover, these authors proposed that activation of β_2_-AR/cPLA_2_ signaling may compensate for depressed cAMP signaling [**4**]. Interestingly, in both of these studies by Pavoine et al. (1999) and Ait-Mamar et al., (2005) cardiomyocytes were cultured on LMN, supporting our findings that cell attachment to LMN may be responsible for inhibition of PKA and activation of β_2_-AR/cPLA_2_ signaling. However, the mechanism by which PKA inhibition activates β_2_-AR/cPLA_2_ signaling is not clear.

Our initial experiments indicated that in atrial myocytes β_2_-AR activation of cPLA_2_ is Ca^2+^-dependent and mediated via ERK1/2 signaling [**3**]. This is consistent with studies in embryonic chick ventricular myocytes (cultured on LMN) in which β_2_-AR stimulation acts via ERK1/2 signaling to activate cPLA_2_ [**6**]. Moreover, in a variety of cell systems Raf-1 activates downstream ERK1/2 and PKA inhibits Raf-1 [**7, 8]**. Therefore, inhibition of PKA should remove inhibition of Raf-1, thereby allowing β_2_-AR stimulation to act via Raf-1/MEK/ERK1/2 signaling. Moreover, protein kinase C (PKC) activates Raf-1 [9, 10]. In other words, PKA inhibits and PKC activates Raf-1/MEK/ERK1/2 signaling. Based on these considerations we sought to determine whether inhibition of PKA facilitates β_2_-AR stimulation to act via PKC/Raf-1/MEK/ERK1/2 to activate cPLA_2_. These findings may be relevant to the remodeling of β_2_-AR signaling in the failing and/or aging heart, both of which exhibit decreases in adenylate cyclase activity.

## Materials and Methods

### Ethics Statement

The animal and experimental protocols used in this study were approved by the Institutional Animal Care and Use Committee (IACUC) of Loyola University Medical Center, Maywood, IL. IACUC prescribed the rules for the animal care and supervised their enforcement. Animals were obtained from a licensed vendor (R & R Research, Howard City, MI., USA), and housed and fed in our AAALAC approved Comparative Medicine Department. Adult cats of either sex (n = 32 cats) were anesthetized with sodium pentobarbital (50 mg/kg, IP).

### Isolation of atrial myocytes

Once fully anesthetized, a bilateral thoracotomy was performed, and the heart was rapidly excised and mounted on a Langendorff perfusion apparatus. After enzyme (collagenase; type II, Worthington Biochemical) digestion, atrial myocytes were isolated as previously reported [**11**].

### Perforated patch clamp experiments

Electrophysiological recordings from atrial myocytes were performed in the perforated (nystatin) patch whole-cell configuration at room temperature, as previously described [**11**]. L-type Ca^2+^ current (I_Ca,L_) was activated by depolarizing pulses from a holding potential of -40 mV to 0 mV for 200 ms every 5 s and measured in relation to steady-state current. β_2_-AR stimulation was achieved by 0.1 μM zinterol (zint-β_2_-AR), a specific β_2_-AR agonist [**12**]. Agonist was applied for approximately 4 min and the effects on peak I_Ca,L_ amplitude were recorded at the steady-state response.

### Plating of atrial myocytes on LMN coated glass coverslips

Generally, we compared freshly isolated atrial myocytes obtained from the same hearts, as previously described [**3**]; atrial myocytes on uncoated glass cover-slips in the absence of PKA inhibitor (+PKA) and atrial myocytes on uncoated glass coverslips exposed to the specific PKA inhibitor (1 μM) KT5720 (-PKA). Because pharmacological inhibition of PKA elicits signaling mechanisms that are similar to those elicited by LMN-integrin signaling, we performed some experiments on atrial myocytes attached to glass cover-slips coated with laminin (+LMN; 40 ug/ml) for at least 2 hrs, as previously described [11]. Inhibition of PKA by KT5720 typically decreases basal I_Ca,L_ amplitude by 15–20% [**3**], consistent with the relatively high endogenous PKA activity in cat atrial myocytes [**11**]. In addition, a variety of experimental results indicate that atrial cell attachment to LMN is not restoring LMN-mediated signaling somehow lost during the cell isolation procedure. For example, control experiments have shown that atrial myocytes plated on poly-L-lysine, a non-specific substrate for cell attachment, fail to exhibit changes in β-AR signaling similar to cells attached to LMN [**1**]. Moreover, freshly isolated cardiomyocytes not plated on LMN exhibit responses to β-AR stimulation which are similar to multicellular cardiac preparations, i.e. exhibit predominantly β_1_-AR over β_2_-AR signaling [**1**]. However, cell attachment to LMN decreases the β_1_-/β_2_-AR signaling ratio resulting in predominantly β_2_-AR over β_1_-AR signaling [**1]**. Moreover, pharmacological inhibition of PKA in myocytes not attached to LMN mimics the effects of cell attachment to LMN **[3]**.

### Adenoviral infection of atrial myocytes

In some experiments, atrial myocytes were attached to laminin (2h) and then infected (100 moi, 24h) with replication-defective adenovirus (Adv) prior to electrophysiological recording. PKCα was inhibited by infection with an Adv expressing kinase-inactive mouse PKCα [**13**], kindly provided by Dr. Trevor Biden, Garvan Institute of Medical Research, St. Vincent’s Hospital, Sydney Australia. An Adv expressing a dominant-negative (dn) mutant of rabbit PKCε [**14]** was kindly provided by Dr. Peipei Ping, University of California-Los Angeles. Adv expressing wild type, and a non-phosphorylatable mutant of human cPLA_2_ (S515A/S505A double mutant) [**15**] were generously provided by Dr. K.U. Malik, University of Tennessee Health Science Center, Memphis, TN. An Adv expressing a dn mutant of human Raf-1 [**9**] was kindly provided by Dr. Dan Kuppuswamy, Medical University of South Carolina, Charleston, SC. A control Adv expressing nuclear-encoded β-galactosidase (Adv-βgal) was used to control for nonspecific effects of adenoviral infection [**16**]. Adenoviruses were amplified and purified using HEK293 cells, and the multiplicity of infection (moi) for each virus was determined by dilution assay in HEK293 cells grown in 96 well clusters, as previously described [**16**]. Preliminary experiments using 5-bromo-4-chloro-3-indolyl-beta- D-galactopyranoside (X-gal) staining of Adv-βgal infected cells determined that a concentration of 100 moi infected 93±3% (n = 3 expts, 400–700 cells/expt) of cultured myocytes. The IP_3_ affinity trap consists of the ligand binding domain of the rat type 1 IP_3_R. The construction of this vector and subsequent production of adenovirus was previously described in detail [**17**]. Freshly isolated atrial myocytes were infected with Adv-IP_3_ affinity trap or the Adv-βgal (control) for 1 h, followed by 18 h short-term culture at 37°C.

### Western blot

Atrial myocytes were plated on poly-L-lysine, a biologically inactive substrate and therefore represent +PKA myocytes. Atrial myocytes were either untreated or treated with Zinterol, KT5720 alone (PKA inhibitor) and KT combined with Zinterol. A positive control showing ERK1/2 and p38MAPK phosphorylation in A7r5 cells is used after stimulation with 1 μM angiotensin II (5 min). Briefly atrial homogenates were centrifuged at 10,000 *g* for 2 min and the supernatant was collected. The protein concentrations of the samples were determined using the Bradford protein assay (Bio-Rad). Aliquots of the samples (40 μg) were dissolved in a Laemmli Sample buffer containing: 60 mM Tris-HCl, 2% SDS, 20% Glycerol, 5% β-mercaptoethanol, 0.01% bromophenol blue (pH 6.8) and the proteins were separated on a 4–15% Mini-PROTEAN TGX Gel (Bio-Rad). After transfer, the membrane was blocked with TBS-T Buffer containing 20 mM Tris pH 7.5, 150 mM NaCl, 0.05% Tween, and a 5% blocking powder (Bio Rad) at 4°C for 1 hr. The blot was probed with primary and secondary antibodies for phorpho-ERK1/2 and phosphor-p38^MAPK^ that were purchased from cell signaling technologies (Danvers, MA). The blot was subsequently probed with GAPDH primary antibody for 2 hrs, followed by HRP-conjugated goat anti-mouse secondary antibody (1:10,000, Santa Cruz Biotechnology) at 4°C for 1 hr. Specific binding was visualized by chemiluminescence (Immun-Star Western C Kit, Bio-Rad) using a ChemiDoc XRS imager (Bio-Rad Life Science Research, Hercules, CA). The intensities of the bands corresponding to each protein were quantified using ImageLab software (Bio-Rad). The relative intensity for each band was normalized to the intensity of the GAPDH staining.

### Chemicals

Zinterol, AACOCF_3_, GW5074, edelfosine (ET-18-OCH3), chelerythrine, 2-aminoethyl diphenyl borate (2-APB), KT5720, methyl-β-cyclodextrin, ryanodine, thapsigargin (Sigma Chemical).

### Statistics

Data are mean ± standard error (SE) of the mean. Measurements were analyzed using either paired or unpaired Student’s *t* test for significance at P<0.05. Multiple comparisons were performed by ANOVA followed by a Student–Newman–Keuls test with significance at P<0.05.

## Results

### Role of Raf-1 in +PKA and –PKA atrial myocytes

**Fig 1:**
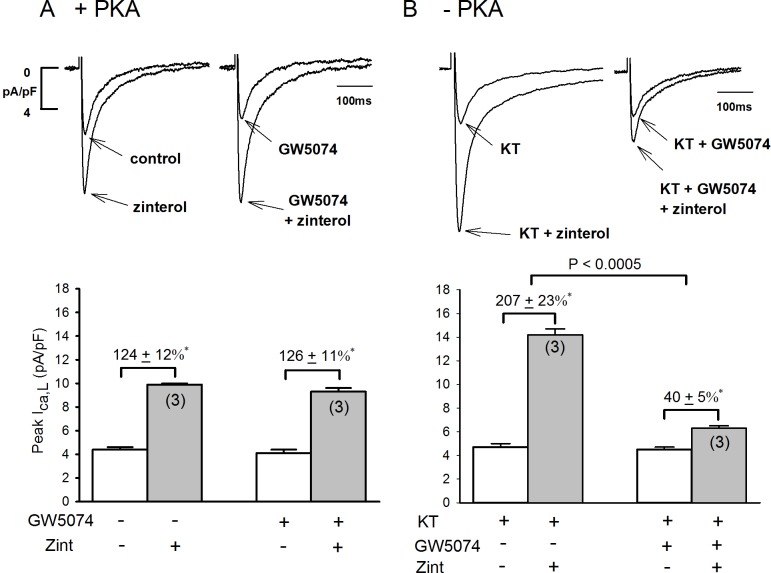
**Effects of Raf-1 inhibition (10 μM GW5074) on zint-β**_**2**_**-AR stimulation of I**_**Ca,L**_ **in +PKA (A), -PKA (B) atrial myocytes.** A; In +PKA myocytes, GW5074 (30 min) had no significant effect on zint-β_2_-AR stimulation of I_Ca,L_. B; In -PKA myocytes, zint-β_2_-AR stimulation of I_Ca,L_ was enhanced compared to control responses (A) and GW5074 significantly inhibited zint-β_2_-AR stimulation of I_Ca,L_. Numbers in parentheses indicate the number of myocytes studied. * = P<0.05.

As shown in **Fig 1**we determined the effects of 10 μM GW5074, a potent Raf-1 inhibitor [**8**] on zint-β_2_-AR stimulation of I_Ca,L_ in +PKA and–PKA atrial myocytes. In +PKA myocytes (A) 0.1 μM zint-β_2_-AR stimulation elicited a typical increase in I_Ca,L_ (124±12%, N = 3). In another group of +PKA myocytes from the same hearts (A), prior exposure to GW5074 for 30 min had no significant effects on basal I_Ca,L_ amplitude (open bar) or zint-β_2_-AR stimulation of I_Ca,L_ (126±11%, N = 3). In contrast, as shown in panel B, control zint-β_2_-AR stimulation of I_Ca,L_ in–PKA myocytes (B; 207±23%, N = 3) was enhanced compared to +PKA myocytes (A; 124±12%, N = 3) due to activation of cPLA_2_ signaling, as previously reported **[4]**. In–PKA myocytes, GW5074 had no effects on basal I_Ca,L_ amplitudes (open bars) but in contrast to +PKA myocytes, GW5074 significantly inhibited zint-β_2_-AR stimulation of I_Ca,L_ (B; 40±5%, N = 3). Additional experiments showed that zint-β_2_-AR stimulation of I_Ca,L_ in +LMN myocytes produced results similar to those found in–PKA myocytes (control; 192±13%, N = 3) and (GW5074; 33±9%, N = 3) (**see S1 Fig for data**).

### Comparison of zint-β_2_-AR induced I_Ca,L_ currents in the Adv-βgal and dn-Raf-1 mutant infected atrial myocytes

**Fig 2:**
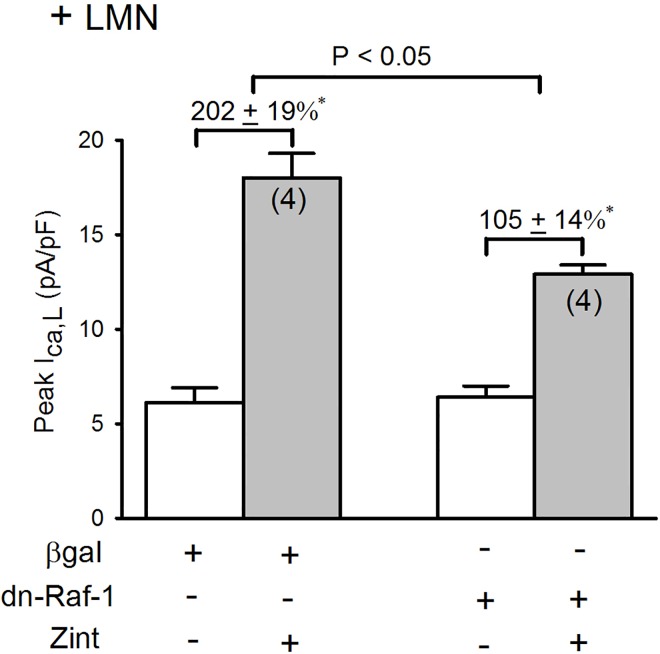
Effects of dn-Raf-1-Adv on zint-β_2_-AR stimulation of I_Ca,L_ in +LMN myocytes. In control +LMN myocytes (βgal) zint-β_2_-AR stimulation elicited a typically enhanced increase in I_Ca,L_. In +LMN myocytes infected with dn-Raf-1-Adv zint-β_2_-AR stimulation of I_Ca,L_ was significantly inhibited compared with controls. Numbers in parentheses indicate that number of cells studied. * = P<0.05.

To further establish the role of Raf-1, we infected atrial myocytes with an Adv that expresses a dn-Raf-1 mutant (generously provided by Dr. Kuppuswamy [9, 18]. Control cells were infected with Adv-βgal. Infected cells were cultured on LMN overnight and therefore represent +LMN myocytes. As shown in **Fig 2A**, in control +LMN myocytes expressing βgal, zint-β_2_-AR stimulation elicited a typically enhanced increase in I_Ca,L_ (202±19%). In +LMN myocytes expressing the dn-Raf-1 mutant, zint-β_2_-AR stimulation of I_Ca,L_ was significantly inhibited (105±14%; P<0.02) compared to control. Together, these findings indicate that Raf-1 signaling plays no role in β_2_-AR stimulation of I_Ca,L_ in freshly isolated atrial myocytes not attached to LMN (**Fig 1A**). However, when PKA is inhibited by either PKA inhibitor or cell attachment to LMN, β_2_-AR stimulation acts via Raf-1 to activate cPLA_2_ and stimulate I_Ca,L_.

### Inhibition of PKA, increases ERK1/2 phosphorylation in atrial myocytes

**Fig 3:**
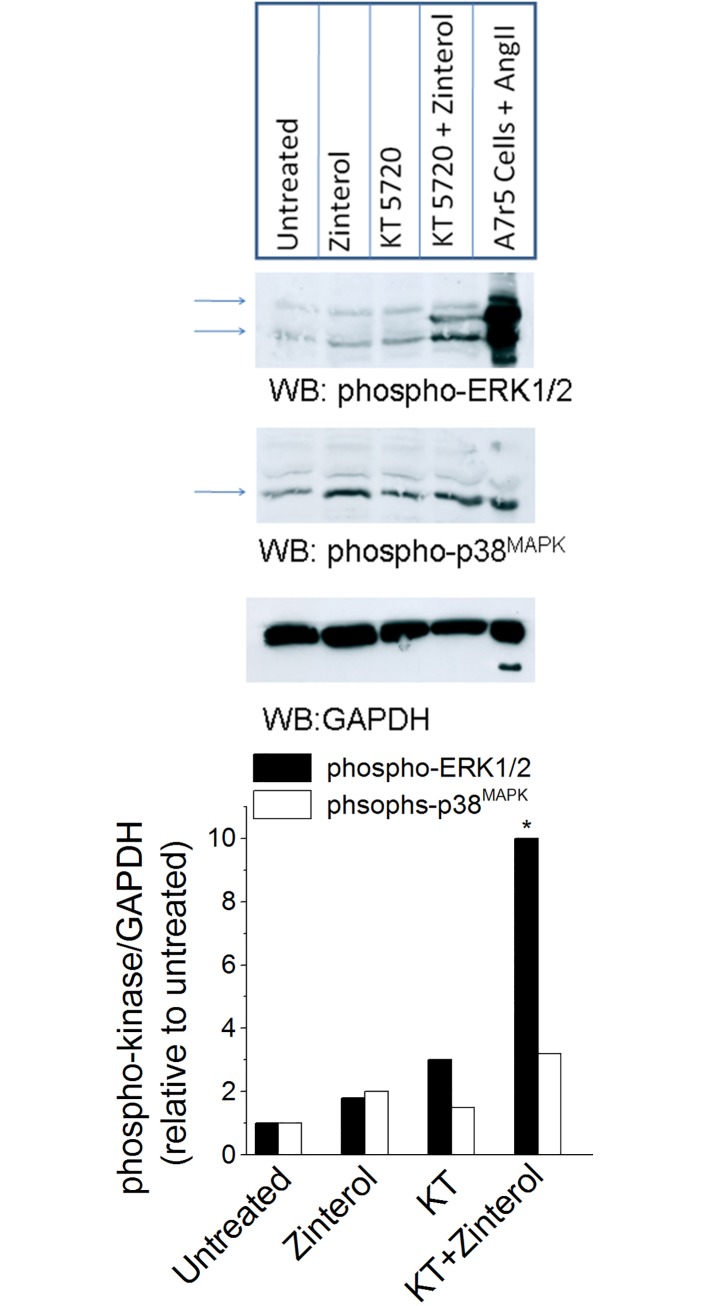
Effect of PKA inhibition on β_2_-AR-medited phosphorylation of ERK1/2. Western blots of atrial cell homogenates show that in control -PKA myocytes zinterol modestly increased ERK1/2 phosphorylation (solid bars). Inhibition of PKA by 1 μM KT5720 (KT) increased basal ERK/1/2 phosphorylation. In the presence of KT5720, zinterol prominently stimulates ERK1/2 phosphorylation. Zinterol only modestly stimulated p38MAPK phosphorylation (open bars), and without significant effect by KT5720. The last lane on the Western blot is a positive control showing ERK1/2 and p38MAPK phosphorylation in A7r5 cells stimulated by 1 μM angiotensin II (5 min). Similar results were obtained in a total of 3 experiments. * = P<0.05.

It is well established that Raf-1 activates downstream MEK/ERK1/2 signaling **[7, 8]**. Moreover, our previous results indicated that inhibition of MEK/ERK1/2 signaling by U0126 inhibited zint-β_2_-AR stimulation cPLA_2_ signaling **[3]**. Therefore, inhibition of PKA should activate β_2_-AR stimulation of ERK1/2 phosphorylation. As shown in **Fig 3**, we performed Western blots and probed for zint-β_2_-AR-mediated ERK1/2 and p38^MAPK^ phosphorylation in the absence (+PKA) and presence of PKA inhibitor (–PKA). Atrial myocytes were plated on poly-L-lysine, a biologically inactive substrate and therefore represent +PKA myocytes. As shown in **Fig 3**in control +PKA myocytes zint-β_2_-AR stimulation modestly increased ERK1/2 phosphorylation (solid bars). Exposure of +PKA myocytes to the PKA inhibitor (1 μM) KT5720, (–PKA myocytes) further increased ERK/1/2 phosphorylation, suggesting that inhibition of basal PKA activity removes PKA-induced inhibition of basal ERK1/2 phosphorylation. In–PKA myocytes, zint-β_2_-AR stimulation prominently stimulated ERK1/2 phosphorylation. Moreover, zint-β_2_-AR stimulation only modestly stimulated p38^MAPK^ phosphorylation (open bars), and was not significantly affected by KT5720. The last lane on the Western blot is a positive control showing ERK1/2 and p38^MAPK^ phosphorylation in A7r5 cells stimulated by 1μM angiotensin II (5 min). In conjunction with our previous findings **[3]**, the present results indicate that inhibition of PKA preferentially activates β_2_-AR stimulation of ERK1/2 signaling.

### Role of PLC in the enhanced zint-β_2_-AR induced I_Ca,L_ currents in –PKA atrial myocytes

**Fig 4:**
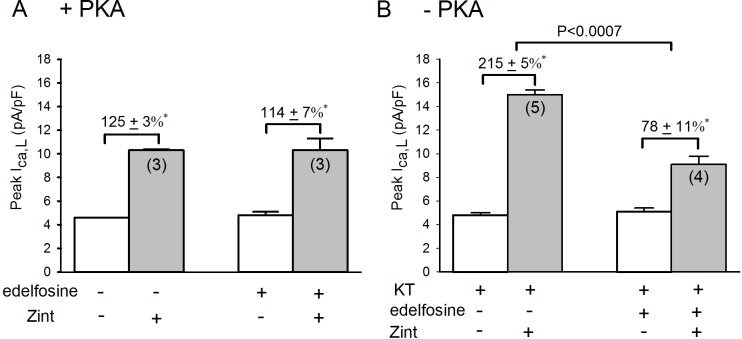
**Effects of 0.5 μM edelfosine on zint-β**_**2**_**-AR stimulation of I**_**Ca,L**_ **in +PKA (A), -PKA(B) and atrial myocytes.** A; in control +PKA myocytes edelfosine had no significant effects on zint-β_2_-AR stimulation of I_Ca,L_. B; in -PKA myocytes zint-β_2_-AR stimulation elicited a typically enhanced increase in I_Ca,L_ compared to controls (A) and edelfosine significantly inhibited zint-β_2_-AR stimulation of I_Ca,L_. Numbers in parentheses indicate the number of myocytes studied. * = P<0.05.

It is well established that Raf-1 activates downstream MEK/ERK1/2 signaling **[7, 8]**. Moreover, our previous results indicated that inhibition of MEK/ERK1/2 signaling by U0126 inhibited zint-β_2_-AR stimulation of cPLA_2_ signaling **[3]**. This raises the question of how β_2_-AR stimulation activates Raf-1. In a variety of cell systems [**10**]**,** including cardiac muscle [**9**], protein kinase C (PKC) activates Raf-1/MEK/ERK1/2. Receptor-mediated activation of phospholipase C (PLC) and the hydrolysis of phospholipids results in the production of diacylglycerol and stimulation of PKC isoenzymes. Therefore, as shown in **Fig 4**we determined the effects of 0.5 μM edelfosine, a specific inhibitor of phospholipase C [**19**], on zint-β_2_-AR stimulation of I_Ca,L_ in +PKA and–PKA myocytes. In control cells (A), edelfosine had little effect on basal I_Ca,L_ amplitude (open bars) or zint-β_2_-AR stimulation of I_Ca,L_ (zint, 125±3% vs edelfosine, 114±7%). In–PKA myocytes (B) zint-β_2_-AR stimulation elicited a typically enhanced increase in I_Ca,L_ (215±5%) compared to control responses (A) due to activation of cPLA_2_ [**3**]. In contrast to +PKA myocytes, edelfosine now significantly inhibited zint-β_2_-AR stimulation of I_Ca,L_ (78±11%; P<0.0005). Additional experiments in +LMN myocytes showed results similar to those found in–PKA myocytes, i.e. zint, 204±2% vs edelfosine, 84±8%; P<0.03 (**see S2 Fig for data**). In other words, inhibition of PKA allows β_2_-AR stimulation to act via PLC to stimulate cPLA_2_ signaling.

### Role of PKC in the enhanced zint-β_2_-AR induced I_Ca,L_ currents in –PKA atrial myocytes

**Fig 5:**
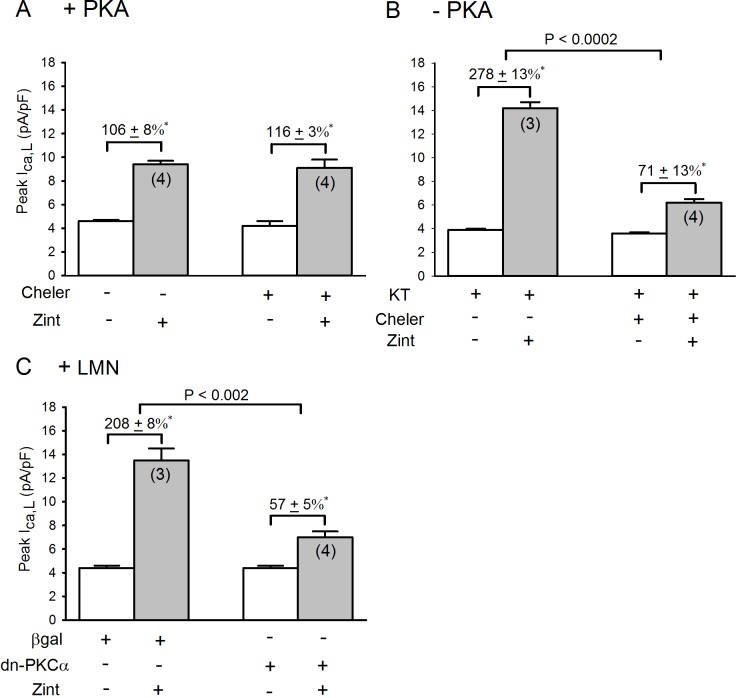
**Effects of 4 μM chelerythrine (cheler) on zint-β**_**2**_**-AR stimulation of I**_**Ca,L**_ **in +PKA(A) and -PKA (B) myocytes and effects of dn-PKCα-Adv (C) on zint-β**_**2**_**-AR stimulation of I**_**Ca,L**_ **in +LMN myocytes.** A; in control +PKA myocytes, chelerythrine had no significant effects on zint-β_2_-AR stimulation of I_Ca,L_. B; in—PKA myocytes, zint-β_2_-AR stimulation elicited a typically enhanced in increase in I_Ca,L_ compared to controls (A) and chelerythrine significantly inhibited zint-β_2_-AR stimulation of I_Ca,L_. C; in control +LMN myocytes (βgal) zint-β_2_-AR stimulation of I_Ca,L_ was typically enhanced compared to controls (A). In +LMN myocytes infected with dn-PKCα-Adv, zint-β_2_-AR stimulation of I_Ca,L_ was significantly inhibited. Numbers in parentheses indicate that number of cells studied. * = P<0.05.

Because activation of PLC produces diacylglycerol and activation of PKC we next determined the effects of 4 μM chelerythrine, an inhibitor of Ca^2+^-dependent and Ca^2+^ -independent PKCs **[20]** on zint-β_2_-AR stimulation of I_Ca,L_ in +PKA and–PKA atrial myocytes. As shown in **Fig 5,** in +PKA myocytes (panel A), chelerythrine had no significant effects on basal I_Ca,L_ amplitude (open bars) or zint-β_2_-AR stimulation of I_Ca,L_ (control,106±8% vs cheler, 116±3%). In–PKA myocytes (panel B) zint-β_2_-AR stimulation elicited a typically enhanced increase in I_Ca,L_ (278±13%) compared to control (panel A) due to activation of cPLA_2_ **[3]**. In contrast to +PKA myocytes, chelerythrine now significantly inhibited zint-β_2_-AR stimulation of I_Ca,L_ (71±13%; P<0.002). These findings indicate PKC signaling plays no role in β_2_-AR stimulation of I_Ca,L_ in freshly isolated atrial myocytes. However when PKA is inhibited β_2_-AR stimulation acts via PKC to stimulate cPLA_2_, consistent with β_2_-AR stimulation of PLC.

In another approach we infected atrial myocyte with an Adv expressing a dominant-negative mutant of Ca^2+^-dependent PKCα (dn-PKCα). Control cells were infected with Adv-βgal. Infected cells were cultured overnight on LMN and therefore represent +LMN myocytes. As shown in **Fig 5C**, in control +LMN myocytes (Adv-βgal) zint-β_2_-AR stimulation elicited a typically enhanced increase in I_Ca,L_ (208±8%) compared to +PKA myocytes (panel A). In +LMN myocytes expressing the dn-PKCα mutant, zint-β_2_-AR stimulation of I_Ca,L_ was significantly inhibited (control, 208±8% vs dn-PKCα, 57±5%; P<0.002). Similar experiments performed on +LMN myocytes expressing a dn-PKCε mutant showed no differences with control +LMN (β-gal) myocytes (control, 158±21% vs dn-PKCε, 165±31%, N = 4). These findings provide additional support for the idea that β_2_-AR stimulation acts via Ca^2+^-dependent PKCα to activate cPLA_2_.

### Role of cPLA2 in the enhanced zint-β_2_-AR induced I_Ca,L_ currents in LMN plated atrial myocytes

**Fig 6:**
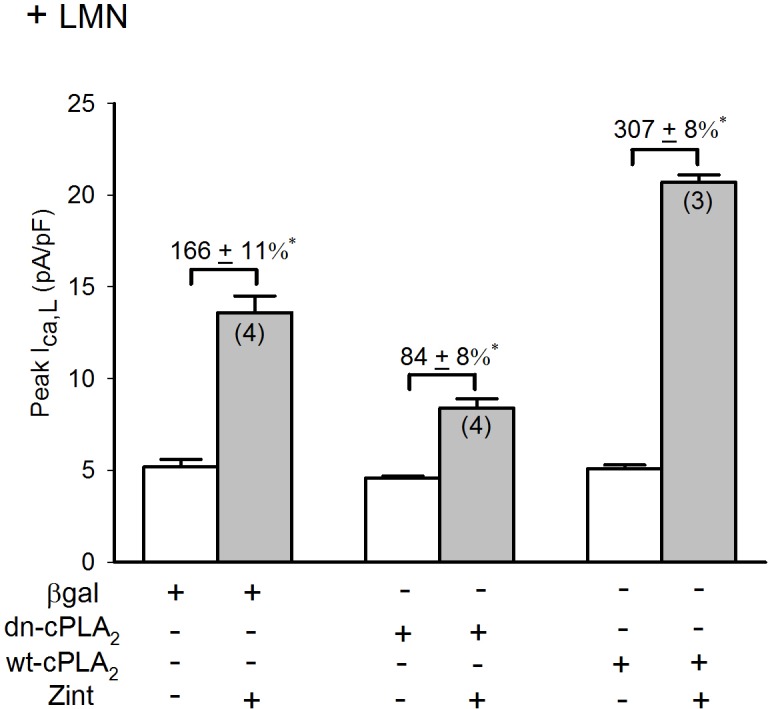
Effects of dn-cPLA_2_^S515A/S505A^ and wt-cPLA_2_ on zint-β_2_-AR stimulation of I_Ca,L_ in +LMN myocytes. Compared to control +LMN myocytes (βgal), zint-β_2_-AR stimulation of I_Ca,L_ was significantly inhibited and enhanced in myocytes overexpressing dn-cPLA_2_^S515A/S505A^ and expressing wt-cPLA_2_, respectively. Numbers in parentheses indicate that number of cells studied. * = P<0.05.

Activation of cPLA_2_ requires phosphorylation at serine sites S505 and S515 [**21–23**]. We therefore determined the effect of β_2_-AR stimulation in +LMN myocytes infected with Adv that either expressed a non-phosphorylatable dominant-negative cPLA_2_ (dn-cPLA_2_^S505A/S515A^) mutant, wild-type cPLA_2_ (wt-cPLA_2_) or βgal as control. Infected atrial myocytes were cultured on LMN overnight and therefore represent +LMN myocytes. **Fig 6**shows that in control +LMN myocytes expressing βgal, β_2_-AR stimulation elicited a typical increase in I_Ca,L_ (166±11%). In +LMN myocytes expressing the double dn-cPLA_2_^S505A/S515A^ mutant β_2_-AR stimulation of I_Ca,L_ was significantly attenuated (84±8%) compared to control. In +LMN myocytes overexpressing wt-cPLA_2_, β_2_-AR stimulation of I_Ca,L_ was significantly enhanced (307±8%) compared to either control or dn-cPLA_2_^S505A/S515A^. These results confirm that cell attachment to LMN activates β_2_-AR/cPLA_2_ signaling and further indicates that β_2_-AR stimulation of cPLA_2_ requires serine phosphorylation at one or both sites.

### Role of IP3 receptor in the enhanced zint-β_2_-AR induced I_Ca,L_ currents in -PKA atrial myocytes

**Fig 7:**
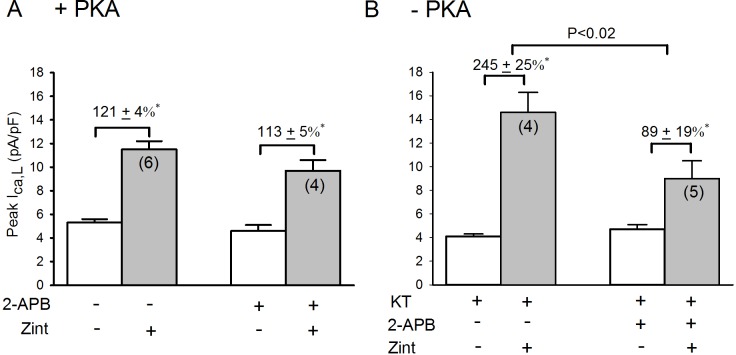
**Effects of 2 μM 2-APB on zint-β**_**2**_**-AR stimulation of I**_**Ca,L**_ **in +PKA (A), -PKA atrial myocytes.** A; in control +PKA myocytes, 2-APB had no significant effects on zint-β_2_-AR stimulation of I_Ca,L_. B; in -PKA myocytes, zint-β_2_-AR stimulation elicited a typically enhanced increase in I_Ca,L_ compared to controls (A) and 2-APB significantly inhibited zint-β_2_-AR stimulation of I_Ca,L_. Numbers in parentheses indicate the number of myocytes studied. * = P<0.05.

Our previous work showed that β_2_-AR stimulation of cPLA_2_ signaling is dependent on intracellular Ca^2+^ [**3**]. The fact that β_2_-AR stimulation acts via PLC to activate cPLA_2_ (**Fig 3**) suggests the potential involvement of IP_3_-mediated Ca^2+^ signaling. We therefore determined the effects of 2 μM 2-APB, a putative IP_3_ receptor (IP_3_R) blocking agent [**24**], on β_2_-AR stimulation of I_Ca,L_ in +PKA and–PKA myocytes. As shown in **Fig 7A**, in +PKA myocytes 2-APB had no significant effect on basal I_Ca,L_ (open bars) or zint-β_2_-AR stimulation of I_Ca,L_ (control, 121±4% vs 2-APB, 113±5%). In–PKA myocytes (B) zint-β_2_-AR stimulation elicited a typically enhanced increase in I_Ca,L_ compared to control responses (panel A). In contrast to +PKA myocytes, 2-APB significantly inhibited zint-β_2_-AR stimulation of I_Ca,L_ (control, 245±25% vs 2-APB, 89±19%; P<0.02). Similar results were obtained in +LMN myocytes where zint-β_2_-AR stimulation of I_Ca,L_ also was significantly blocked by 2-APB (control, 186±26%; N = 4 vs 87±7%; N = 4, P<0.01) (**see S3 Fig online data**).

**Fig 8:**
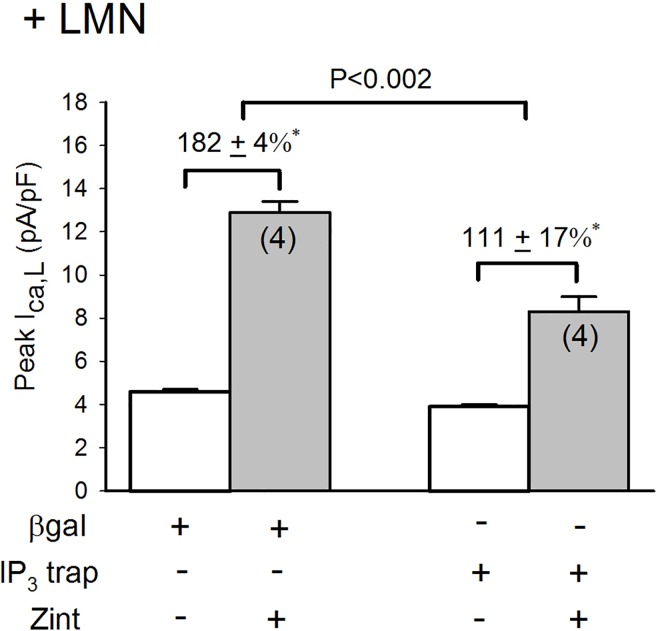
Effect of adenovirus IP_3_ affinity trap on zint-β_2_-AR stimulation of I_Ca,L_ in +LMN myocytes. Compared with control +LMN myocytes (βgal), zint-β_2_-AR stimulation of I_Ca,L_ was significantly inhibited in +LMN myocytes infected with adenovirus expressing the IP_3_ affinity trap. Numbers in parentheses indicate the number of myocytes studied. * = P<0.05.

In another approach, we infected atrial myocytes with an adenovirus that expresses an IP_3_ affinity trap which binds to IP_3_ in the cytosol and thereby inhibits IP_3_-mediated Ca^2+^ signaling by preventing IP_3_ from reaching and activating the IP_3_ receptor (IP_3_R) [**17**]. Cells were cultured overnight on LMN and therefore represent +LMN myocytes. As shown in **Fig 8,** in control +LMN myocytes expressing βgal, zint-β_2_-AR stimulation elicited a typical increase in I_Ca,L_ (182±4%). However, in +LMN myocytes expressing the IP_3_ trap zint-β_2_-AR stimulation of I_Ca,L_ (111±17%) was significantly inhibited compared to controls (P<0.002). Together, these findings suggest that IP_3_R signaling plays no role in zint-β_2_-AR stimulation of I_Ca,L_ in freshly isolated atrial myocytes. However, when PKA is inhibited by either PKA inhibitor (**Fig 7**) or cell attachment to LMN (**Fig 8**), β_2_-AR stimulation of cPLA_2_ is dependent on IP_3_-mediated Ca^2+^ signaling.

### Role of IP3 receptors located on SR or nuclear envelope in the enhanced zint-β_2_-AR induced I_Ca,L_ currents in -PKA atrial myocytes

**Fig 9:**
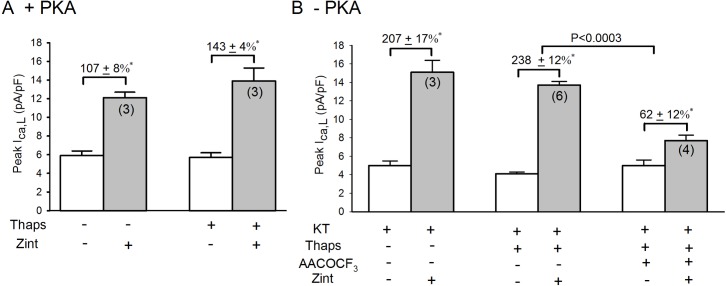
**Effects of 5 μM thapsigargin (10 min; Thaps) on zint-β**_**2**_**-AR stimulation of I**_**Ca,L**_ **in +PKA(A) and -PKA (B) myocytes.** A; in +PKA myocytes, compared to control responses, thapsigargin slightly enhanced zint-β_2_-AR stimulation of I_Ca,L_. B; in -PKA myocytes, zint-β_2_-AR stimulation elicited a typically enhanced increase in I_Ca,L_ compared to control (A) that was not prevented by treatment with thapsigargin. The addition of 10 μM AACOCF_3_ (+ thaps) significantly inhibited zint-β_2_-AR stimulation of I_Ca,L_ indicating that thapsigargin did not prevent zint-β_2_-AR stimulation of cPLA_2_. Numbers in parentheses indicate the number of myocytes studied. * = P<0.05.

IP_3_Rs are thought to be located primarily on the sarcoplasmic reticulum (SR) and nuclear envelope membranes. Because the SR and nuclear envelope membranes are highly interconnected, inhibition of SR Ca^2+^ uptake by thapsigargin depletes intracellular Ca^2+^ stores from both sites [**25**]. Therefore, in **Fig 9**we determined whether depletion of SR and nuclear Ca^2+^ by 5 μM thapsigargin (10 min; Thaps) inhibits β_2_-AR stimulation of I_Ca,L_ in +PKA (A) and–PKA (B) myocytes. In +PKA myocytes (A) thapsigargin had no effect on basal I_Ca,L_ amplitude (open bars). Compared to control responses (107±8%), thapsigargin slightly enhanced zint-β_2_-AR stimulation of I_Ca,L_ (143±4%), although the change was not statistically significant. This modest increase in β_2_-AR stimulation of I_Ca,L_ is consistent with the effects of thapsigargin to inhibit SR Ca^2+^ release and thereby inhibit Ca^2+^-mediated inactivation of I_Ca,L_. In fact, separate experiments showed that thapsigargin abolished SR Ca^2+^ transients (data not shown). In–PKA myocytes (B), zint-β_2_-AR stimulation of I_Ca,L_ (207±17%) was typically enhanced compared to control responses (107±8%) obtained in +PKA myocytes (A). Interestingly, in–PKA myocytes treated with thapsigargin β_2_-AR stimulation of I_Ca,L_ (238±12%) was still enhanced. In other words, depletion of Ca^2+^ from SR and nuclear membranes failed to prevent PKA inhibition from enhancing β_2_-AR signaling. Moreover, the last column in **Fig 9B** shows that in–PKA myocytes treated with thapsigargin, AACOCF_3_ (cPLA2 inhibitor) significantly inhibited zint-β_2_-AR stimulation of I_Ca,L_ (62±12%), indicating that the enhanced response to zint-β_2_-AR stimulation was in fact due to activation of cPLA_2_ signaling and that depletion of SR and nuclear Ca^2+^ stores failed to prevent β_2_-AR/cPLA_2_ signaling. Similar results were obtained when Ca^2+^ stores were depleted by treatment (10 min) with 10 μM ryanodine (data not shown). These results indicate that IP_3_-dependent Ca^2+^ signaling is not mediated via IP_3_Rs located on SR or nuclear envelope membranes.

### Role of IP3 receptors located in caveolae in the enhanced zint-β_2_-AR induced I_Ca,L_ currents in -PKA atrial myocytes

**Fig 10:**
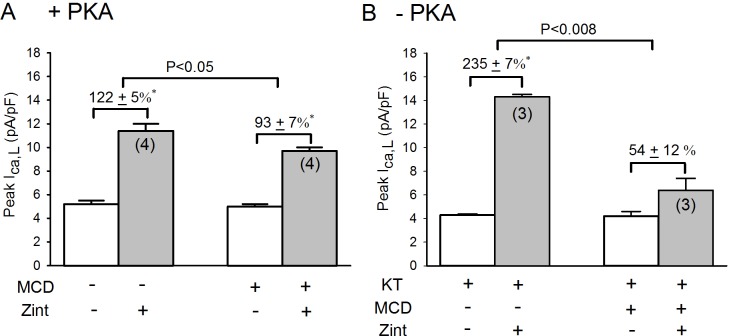
**Effect of 2 mM methyl-β-cyclodextrin (MCD; 30 min) on zint-β**_**2**_**-AR stimulation of I**_**Ca,L**_ **in +PKA (A) and -PKA (B) myocytes.** A; in +PKA myocytes, MCD significantly decreased zint-β_2_-AR stimulation of I_Ca,L_ compared to control responses (-24%). B; in—PKA myocytes, zint-β_2_-AR stimulation elicited a typically enhanced increase in I_Ca,L_ compared to +PKA myocytes (A) and the effect of MCD to decrease zint-β_2_-AR stimulation of I_Ca,L_ was enhanced (-77%) compared to +PKA myocytes (A). Numbers in parentheses indicate the number of myocytes studied. * = P<0.05.

Alternatively, IP_3_R protein is present in caveolae **[26, 27]**, which are abundant in atrial myocytes [**28**]. We therefore determined the effects of 10 mM methyl-β-cyclodextrin (MCD; 30 min), an agent that disrupts caveolae formation [**29**], on zint-β_2_-AR stimulation of I_Ca,L_ in +PKA and–PKA myocytes. As shown in **Fig 10A**, in +PKA myocytes MCD caused a modest but significant inhibition of zint-β_2_-AR stimulation of I_Ca,L_ (control,122±5% vs MCD, 93±7%; P<0.05), representing a 24% decrease. This is consistent with the idea that β_2_-ARs are normally localized to caveolae [**30**]. However, in–PKA myocytes (B) MCD elicited a significantly larger inhibition of zint-β_2_-AR stimulation of I_Ca,L_ (control, 235±7% vs MCD, 54±12%; P<0.008), representing a 77% decrease. The fact that MCD elicited a significantly larger inhibition of β_2_-AR signaling in–PKA compared to +PKA myocytes supports the idea that the IP_3_Rs that are essential for β_2_-AR/cPLA_2_ signaling are localized to the caveolae.

## Discussion

We previously reported that in atrial myocytes inhibition of adenylate cyclase/PKA by either cell attachment to LMN or inhibition of PKA in cells not attached to LMN activates β_2_-AR/cPLA_2_ signaling **[2, 3]**. The present study extends those findings by showing that when PKA is inhibited, β_2_-AR stimulation acts via PKCα/Raf-1/MEK/ERK1/2 and IP_3_-dependent Ca^2+^ signaling to activate cPLA_2_.

In the present experiments, we showed that inhibition of PKA prominently stimulates β_2_-AR-mediated phosphorylation of ERK1/2, consistent with β_2_-AR stimulation of I_Ca,L_ via MEK/ERK1/2 signaling **[3]**. Moreover, inhibition of Raf-1 by either GW5074 or adenoviral expression of a dn-Raf-1 mutant significantly inhibited β_2_-AR stimulation of I_Ca,L_ in–PKA or +LMN myocytes but failed to affect β_2_-AR signaling in +PKA myocytes. These findings can be explained by the fact that PKA inhibits Raf-1 and Raf-1 activates MEK/ERK1/2 signaling **[7, 31, 32]**. Because atrial myocytes normally exhibit relatively high endogenous levels of adenylate cyclase/PKA activity [**11**], Raf-1 is normally inhibited and therefore β_2_-AR stimulation is unable to activate Raf-1/MEK/ERK1/2 signaling in +PKA myocytes. However, when PKA is inhibited (–PKA or +LMN myocytes) β_2_-AR stimulation acts via Raf-1/MEK/ERK1/2 to activate cPLA_2_. The present experiments also show that adenoviral overexpression of wt-cPLA_2_ or expression of a dn-cPLA_2_^S505A/S515A^ mutant in +LMN myocytes significantly enhanced or inhibited, respectively, β_2_-AR stimulation of I_Ca,L_ compared to control +LMN myocytes expressing βgal. In various cell systems including vascular smooth muscle and fibroblasts ERK1/2 phosphorylates cPLA_2_ at S505 and S515 **[15, 21–23].** In cardiac ventricular myocytes (cultured on LMN) β_2_-AR stimulation acts via ERK1/2 and p38^MAPK^ to phosphorylate cPLA_2_ at S505 [**6**].

In atrial myocytes, β_2_-ARs are coupled to both Gs- and Gi-mediated signaling pathways [**12**]. We [**3**] and others [**4**] have reported that β_2_-ARs act via pertussis toxin-sensitive G_i_ to activate cPLA_2_. In various cell systems, including rat ventricular myocytes [**33**] activation of PKA phosphorylates β_2_-ARs and thereby switches β_2_-AR coupling from G_s_-mediated adenylate cyclase to G_i_-mediated ERK1/2 signaling [**34, 35]**. Moreover, inhibition of PKA by H-89 inhibited β_2_-AR/G_i_-mediated ERK1/2 signaling. These findings are not consistent with the present results, which indicate that in adult atrial myocytes inhibition of PKA by H-89 or KT5720 activates (rather than inhibits) β_2_-AR/G_i_-mediated ERK1/2 signaling [**3**]. However, our findings are consistent with reports that PKA inhibits Raf-1 and its downstream MEK/ERK1/2 signaling pathway [**7, 31, 32]**. Therefore, in atrial myocytes inhibition of PKA removes Raf-1 inhibition and thereby activates β_2_-AR/G_i_ stimulation of Raf-1/MEK/ERK1/2 signaling.

The present results also indicate that inhibition of PLC (edelfosine) or PKC (chelerythrine or dn-PKCα) significantly inhibited β_2_-AR stimulation of I_Ca,L_ in–PKA or +LMN myocytes but not in +PKA myocytes. Moreover, in +LMN myocytes expression of dn-PKCα significantly inhibited β_2_-AR signaling while expression of dn-PKCε had no effect, indicating that β_2_-ARs act via Ca^2+^-dependent PKCα to activate cPLA_2_. These findings are consistent with reports that PKC activates Raf-1/MEK/ERK1/2 signaling in adult cardiac myocytes [**9, 18]** and that PKC activates cPLA_2_ signaling [**10**].

Our previous findings showed that strong chelation of intracellular Ca^2+^ by BAPTA prevented β_2_-AR stimulation via cPLA_2_. This is consistent with the fact that several of the signaling molecules involved in the proposed β_2_-AR/cPLA_2_ signaling cascade are Ca^2+^-dependent, including PLC, PKCα and cPLA_2_. In the present study, we investigated more specifically which source of intracellular Ca^2+^ is required for β_2_-AR/cPLA_2_ signaling. We found that 2-APB, an agent that inhibits IP_3_-mediated Ca^2+^ release in cat atrial myocytes **[24**] significantly inhibited β_2_-AR stimulation of I_Ca,L_ in both–PKA and +LMN myocytes but not in +PKA myocytes. Moreover, adenoviral expression of an IP_3_ affinity trap which inhibits IP_3_-mediated Ca^2+^ signaling [**17**] resulted in a similar inhibition of β_2_-AR signaling in +LMN myocytes. The fact that 2-APB had no effect on β_2_-AR signaling in control +PKA myocytes and that it exerted effects similar to those of the Adv-IP_3_ affinity trap suggests that 2-APB acted specifically to inhibit IP_3_-mediated Ca^2+^ signaling. Together, these results indicate that β_2_-AR stimulation of cPLA_2_ is dependent on IP_3_-mediated Ca^2+^ signaling. Although IP_3_Rs are typically located on SR and nuclear membranes, the present results showed that thapsigargin, an agent that depletes both SR and nuclear envelope Ca^2+^ stores [**25**] failed to prevent β_2_-AR stimulation of I_Ca,L_ via cPLA_2_. However, disruption of caveolae formation by methyl-β-cyclodextrin elicited a significantly larger inhibition of β_2_-AR stimulation in–PKA myocytes than control +PKA myocytes. We therefore conclude that IP_3_Rs on the SR and nuclear envelope membranes are not involved in the Ca^2+^ signaling required for β_2_-AR stimulation of cPLA_2_. Alternatively, β_2_-AR stimulation of I_Ca,L_ via cPLA_2_ is dependent on IP_3_-mediated Ca^2+^ signaling in caveolae. In endothelial and smooth muscle cells plasmalemmal caveolae contain IP_3_R protein that is speculated to mediate Ca^2+^ influx through the caveolar membrane [**26, 27]**. Moreover, in rat ventricular myocytes (cultured on laminin) β_2_-ARs stimulate translocation of cPLA_2_ to the low density caveolin-3 enriched membrane fraction suggesting that cPLA_2_ translocates to caveolae [**5**]. In fact, lipid rafts i.e. caveolae contain a wide variety of signaling components including β_2_-ARs, G_i_, PKC, Raf-1, ERK1/2, IP_3_Rs **[36**], involved in β_2_-AR/cPLA_2_ signaling.

**Fig 11:**
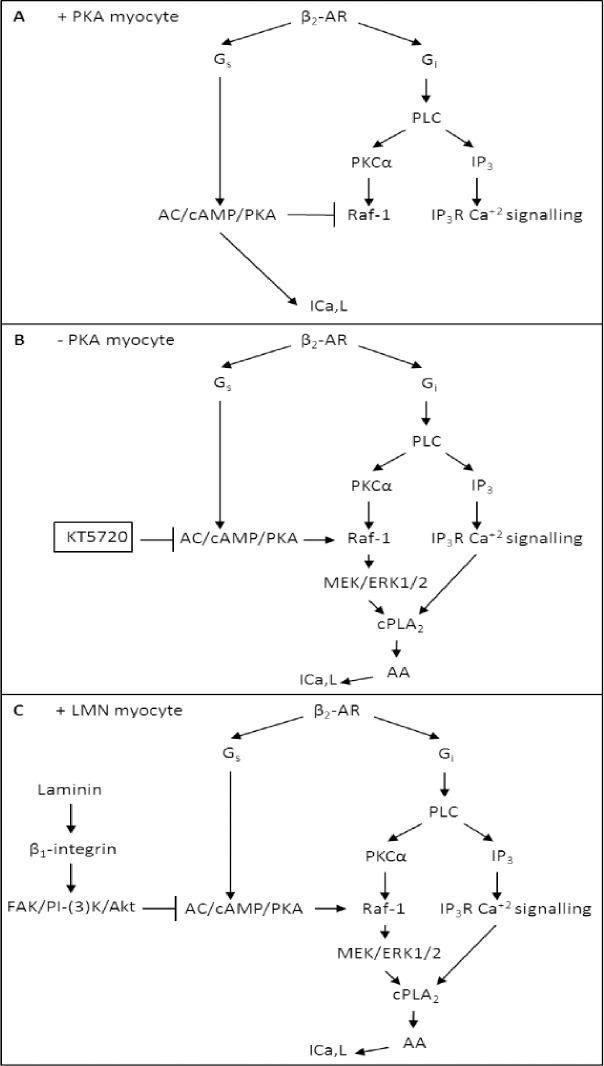
**Schematic summary showing the proposed signaling mechanisms underlying zint-β**_**2**_**-AR stimulation of I**_**Ca,L**_ **in +PKA (A), -PKA (B) atrial myocytes.** A; in +PKA myocytes, zint-β_2_-AR stimulation acts via G_s_ to activate adenylate cyclase (AC)/cAMP-dependent kinase (PKA) which in turn stimulates I_Ca,L_. Both basal and stimulated PKA activity inhibits Raf-1 signaling, thereby preventing zint-β_2_-AR stimulation of I_Ca,L_ via cPLA_2_. B; in cells not attached to LMN, inhibition of PKA by KT5720 removes inhibition of Raf-1. C; cell attachment to LMN acts via β_1_ integrins and FAK/PI-(3)K/Akt signaling to inhibit adenylate cyclase (AC)/cAMP-dependent kinase (PKA) activity, thereby removing inhibition of Raf-1. β_2_-AR stimulation acts via G_i_ to stimulate PLC leading to activation of PKCα and IP_3_R-mediated Ca^2+^ signaling within caveolae. With PKA inhibited, PKCα stimulates Raf-1/MEK/ERK1/2 signaling. Together, ERK1/2 and IP_3_-mediated Ca^2+^ signaling activate cPLA/AA, resulting in stimulation of I_Ca,L_.

We therefore propose (**see Fig 11**) that under normal conditions (A; +PKA myocytes), β_2_-AR/G_s_ stimulation acts via adenylate cyclase (AC)/cAMP/PKA to stimulate I_Ca,L_. The relatively high basal levels of endogenous PKA activity and β_2_-AR-stimulated PKA activity both inhibit Raf-1 signaling, thereby preventing β_2_-AR stimulation from normally activating cPLA_2_. However, inhibition of PKA by either PKA inhibitor (B;–PKA myocytes) or by cell attachment to LMN (C; +LMN myocytes), removes inhibition of Raf-1. Under these conditions, β_2_-AR/G_i_ stimulation of PLC activates PKCα and IP_3_R-mediated Ca^2+^ signaling in caveolae. With PKA inhibited, PKCα is now able to stimulate Raf-1/MEK/ERK1/2 signaling. Therefore, β_2_-AR/G_i_-mediated activation of both ERK1/2 and IP_3_-dependent Ca^2+^ signaling stimulates cPLA_2_/AA. We previously reported that arachidonic acid (AA) stimulates I_Ca,L_ [**3**]. This scenario is consistent with the fact that cPLA_2_ activation requires both elevation of submicromolar intracellular [Ca^2+^] and phosphorylation by various kinases (see also [**21–23**]).

### Conclusions

These findings may have important implications with respect to the aging and/or failing heart, both of which exhibit decreases in adenylate cyclase activity. In both animal models [**37**] and in the human right atrium [**38**], increasing age is associated with a decrease in β_1_-AR function that results from a decrease in adenylate cyclase activity. Likewise, adenylate cyclase activity is depressed in the failing human heart [**39**] and canine pacing-induced heart failure **[40, 41]** and yet β_2_-AR signaling is preserved. Previous studies by Nalli et al suggest that PKA and PKG phosphorylates PLCβ3 in gastric smooth muscle cells and decreases PI hydrolysis **[42]**. The decrease in PI hydrolysis has been suggested to diminish IP_3_-mediated Ca^2+^ release decreasing muscle contraction. Along these lines, the present studies also suggest that feline atrial cardiomyocytes exhibit a similar regulation as that seen in gastric muscle. Our research also suggests that increases in extracellular matrix proteins, i.e. fibrosis, may contribute to decreases in adenylate cyclase/PKA activity in the aging and/or failing heart, which in turn switches β_2_-AR signaling from cAMP/PKA to cPLA_2_/AA. This mechanism may be responsible for the preservation of β_2_-AR signaling while β_1_-AR signaling is depressed. Thus, the diminished PKA activity in cardiac fibrosis may decrease phosphorylation of PLC resulting in an increase in IP hydrolysis, Ca^2+^ release and greater contraction. Indeed, our present results indicate that inhibition of PLC significantly inhibited β_2_-AR stimulation of I_Ca,L_ in–PKA or +LMN myocytes but not in +PKA myocytes. In addition, our results suggest that 2-APB, an agent that inhibits IP_3_-mediated Ca^2+^ release in cat atrial myocytes **[24]** significantly inhibited β_2_-AR stimulation of I_Ca,L_ in both–PKA and +LMN myocytes but not in +PKA myocytes. Together, these results indicate that β_2_-AR stimulation of cPLA_2_ in both–PKA and +LMN myocytes is dependent on PLC/IP_3_-mediated Ca^2+^ signaling. Given that cPLA_2_/AA signaling is a potentially pro-inflammatory mediator, the present findings suggest that fibrosis in the aging and/or failing heart may predispose inflammation and atrial dysfunction. In fact, AA is reported to slow atrial conduction and has been implicated in the development of postoperative atrial fibrillation **[43]**. On the other hand, activation of the Raf/MEK/ERK pathway may be cardioprotective **[44]**. In fact, inhibition of adenylate cyclase/PKA and subsequent activation of Raf-1/MEK/ERK1/2 signaling enhances cardiac resistance to oxidative stress, increases cell survival, and extends lifespan **[45]**. Therefore, inhibition of adenylate cyclase/PKA activity and the remodeling of β_2_-AR signaling to activate Raf/MEK/ERK1/2 may help ameliorate the deterioration of function that occurs in the aging and/or failing atrium. Previous studies have indicated that, beside inhibition of COX, aspirin has COX-independent mechanisms that plays a role in tumor suppression **[46, 47]**. Aspirin reportedly inhibits PI3K/Akt kinase activity in epithelial ovarian cancer cells **[48]**. Aspirin down regulates the expression of PI3K, Akt and ERK in rodent model of acute pulmonary embolism **[49]** and in turn inhibit the release of inflammatory cytokines **[50]**. On the other hand, our studies indicate that laminin acts via FAK/PI-(3)K/Akt signaling to inhibit adenylate cyclase-mediated stimulation of I_Ca,L_. Although, there is no direct evidence suggesting that aspirin or NSAIDs play a role in cardiomyocyte aging, we hypothesize that aspirin or NSAIDs may influence cPLA2/AA pathway and this requires further investigation.

## Supporting Information

S1 Fig Effects of Raf-1 inhibition (10 μM GW5074) on zint-β_2_-AR stimulation of I_Ca,L_ in +LMN atrial myocytes. A; In +LMN myocytes, zint-β_2_-AR stimulation of I_Ca,L_ was enhanced and GW5074 significantly inhibited zint-β_2_-AR stimulation of I_Ca,L_. Numbers in parentheses indicate the number of myocytes studied. * = P<0.004.
(TIF)

S2 Fig Effects of 0.5 μM edelfosine on zint-β_2_-AR stimulation of I_Ca,L_ in +LMN atrial myocytes. A; in +LMN myocytes zint-β_2_-AR stimulation elicited a typically enhanced increase in I_Ca,L_ and edelfosine significantly inhibited zint-β_2_-AR stimulation of I_Ca,L_. Numbers in parentheses indicate the number of myocytes studied. * = P<0.03.
(TIF)

S3 Fig Effects of 2 μM 2-APB on zint-β_2_-AR stimulation of I_Ca,L_ in +LMN atrial myocytes. A; +LMN myocytes, zint-β_2_-AR stimulation elicited a typically enhanced increase in I_Ca,L_ and 2-APB significantly inhibited zint-β_2_-AR stimulation of I_Ca,L_. Numbers in parentheses indicate the number of myocytes studied. * = P<0.01.
(TIF)

## Abbreviations

LMN: laminin
cPLA_2_: cytosolic phospholipase A_2_
AA: arachidonic acid
ERK: extracellular-signal regulated kinase
MEK: mitogen-activated protein kinase kinase
Raf-1 (also known as c-Raf): MAP kinase kinase kinase
PLC: phospholipase C
FAK: focal adhesion kinase
PI-(3)K: phosphatidyinositol-3’ kinase
Akt: protein kinase B
zint-β_2_-AR: zinterol β_2_-adrenergic receptor
I_Ca,L_: L-type Ca^2+^ current
Adv: adenovirus
dn: dominant negative
