# Supplementary figures and images for: Inhibition of cAMP-Dependent PKA Activates β2-Adrenergic Receptor Stimulation of Cytosolic Phospholipase A2 via Raf-1/MEK/ERK and IP3-Dependent Ca2+ Signaling in Atrial Myocytes
Source: PLoS One. 2016 Dec 15;11(12):e0168505. doi: 10.1371/journal.pone.0168505 (PMC5158063; doi:10.1371/journal.pone.0168505)

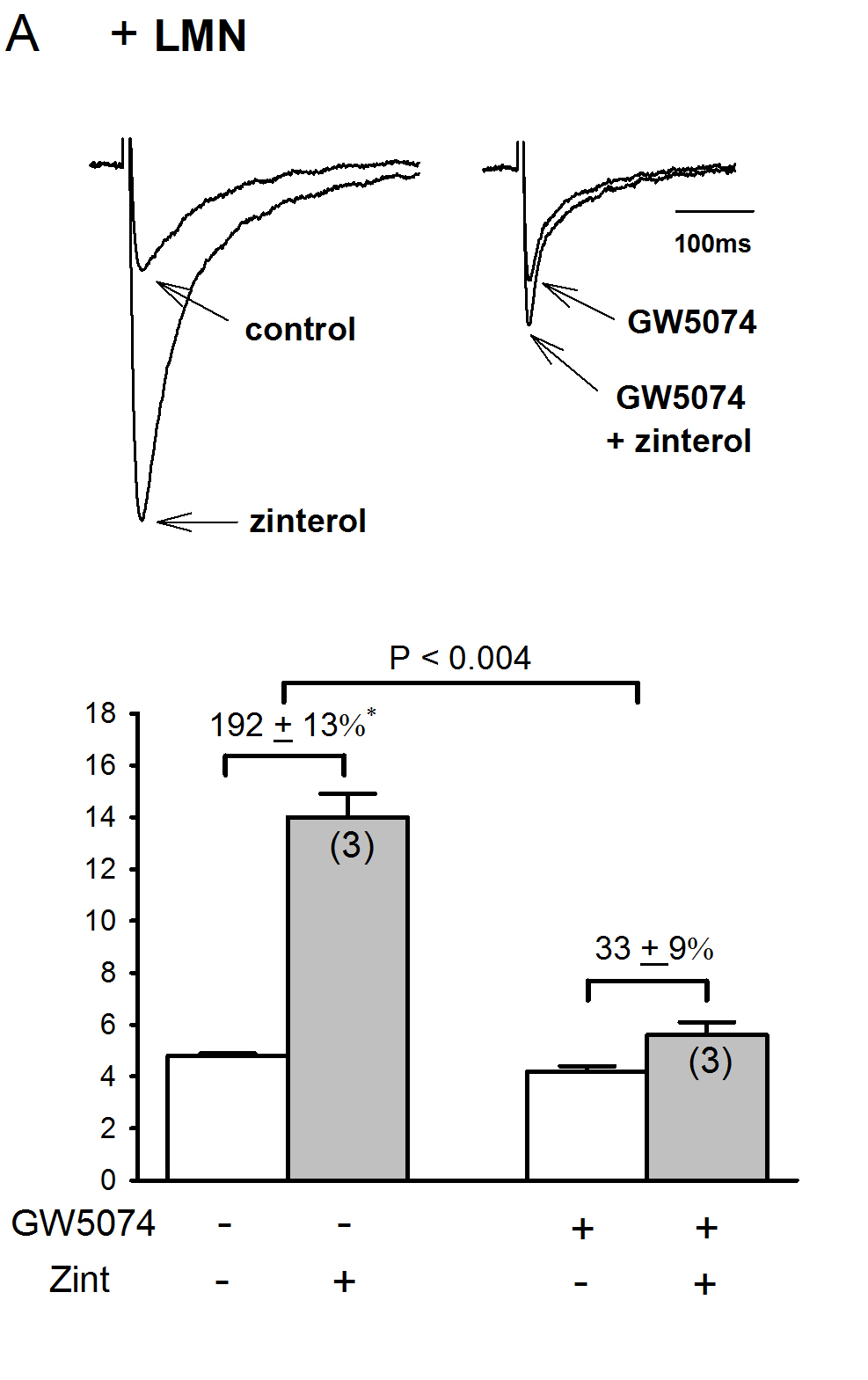

Supplement: S1 Fig — A; In +LMN myocytes, zint-β2-AR stimulation of ICa,L was enhanced and GW5074 significantly inhibited zint-β2-AR stimulation of ICa,L. Numbers in parentheses indicate the number of myocytes studied. * = P<0.004. (TIF) [file pone.0168505.s001.tif]

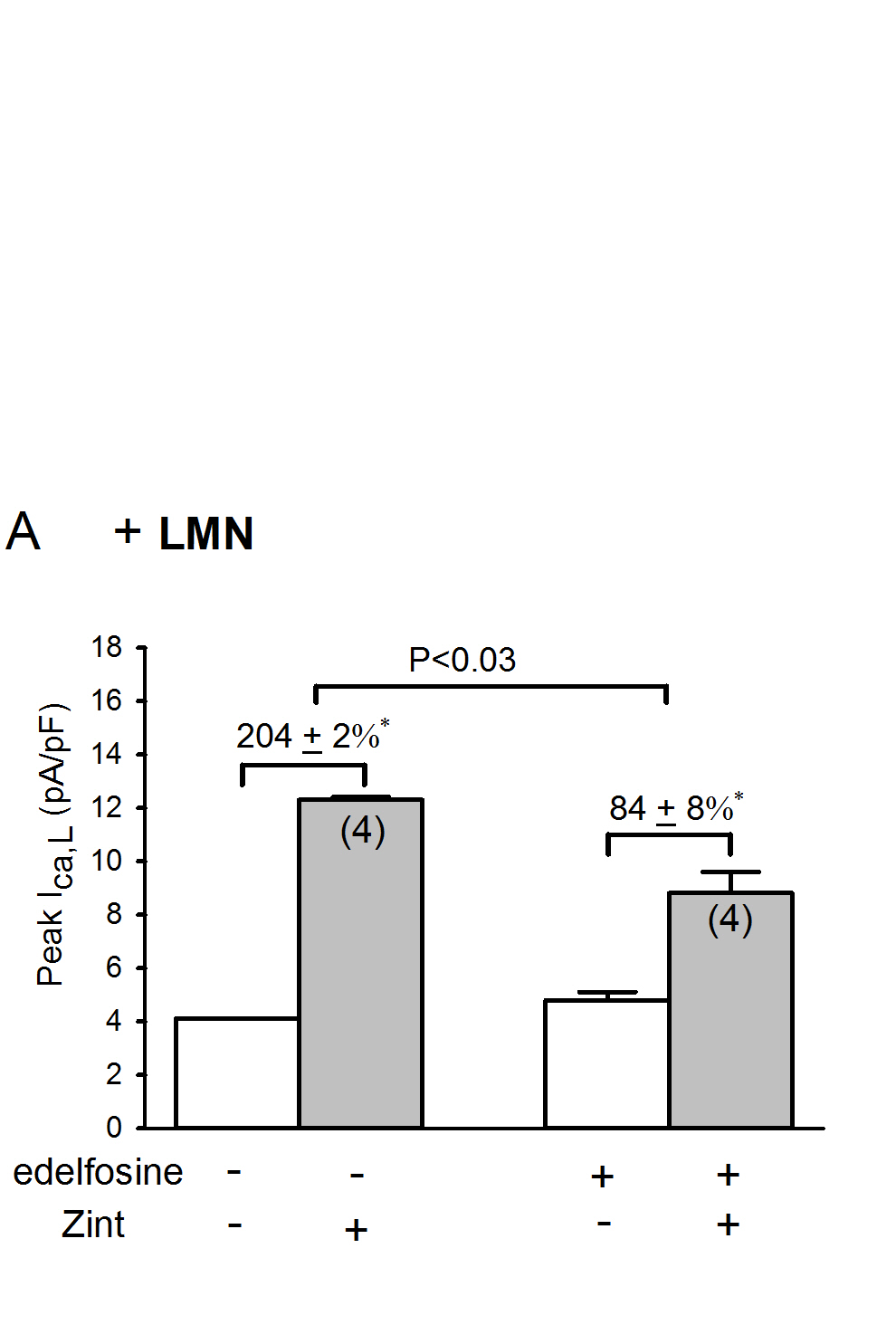

Supplement: S2 Fig — A; in +LMN myocytes zint-β2-AR stimulation elicited a typically enhanced increase in ICa,L and edelfosine significantly inhibited zint-β2-AR stimulation of ICa,L. Numbers in parentheses indicate the number of myocytes studied. * = P<0.03. (TIF) [file pone.0168505.s002.tif]

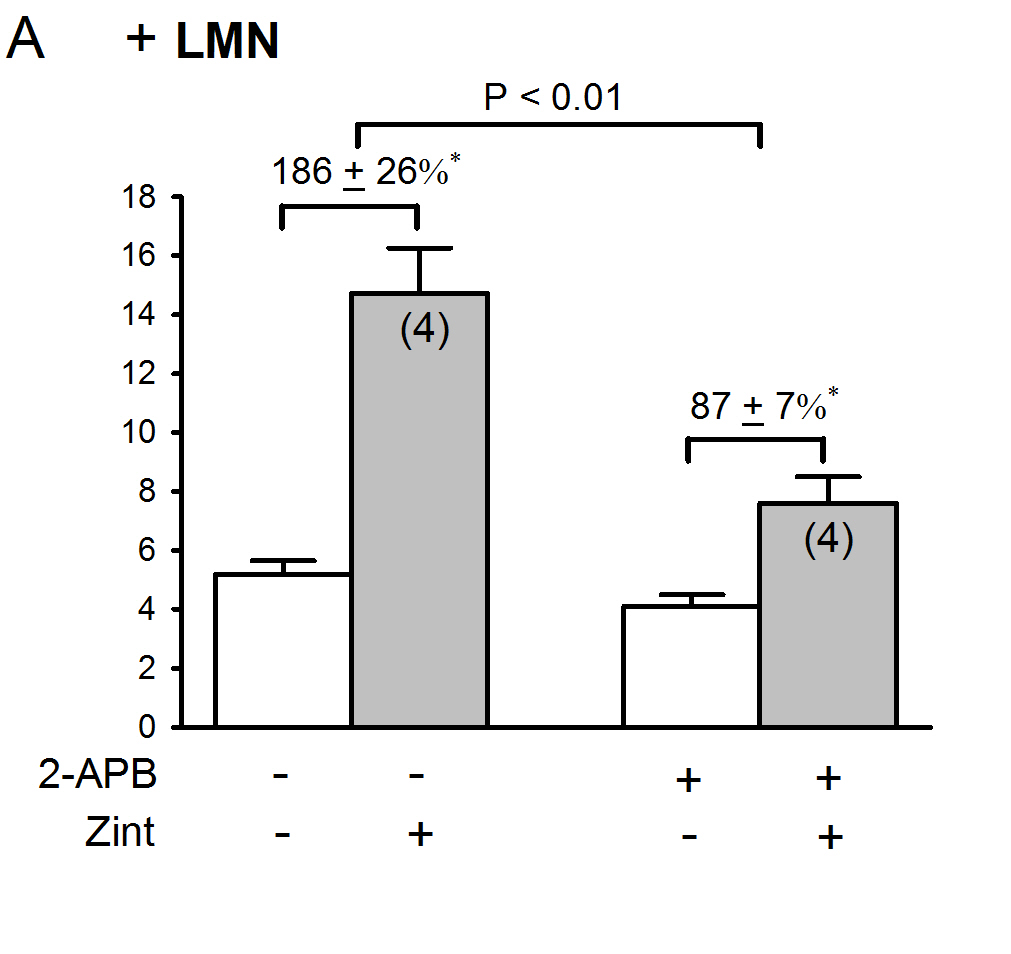

Supplement: S3 Fig — A; +LMN myocytes, zint-β2-AR stimulation elicited a typically enhanced increase in ICa,L and 2-APB significantly inhibited zint-β2-AR stimulation of ICa,L. Numbers in parentheses indicate the number of myocytes studied. * = P<0.01. (TIF) [file pone.0168505.s003.tif]
